# Physical Activity in Osteosarcoma Patients During and Post Therapy: A Single Site Prospective Observational Study

**DOI:** 10.1002/cam4.71674

**Published:** 2026-02-26

**Authors:** Elysia R. Cohen, Clark Andersen, Karen Moody, Maria C. Swartz, Michael C. Robertson, Alakh Rajan, Theresa Honey, Sandra Lugo, Grace Waterman, Keri Schadler

**Affiliations:** ^1^ Division of Pediatrics The University of Texas MD Anderson Cancer Center Houston Texas USA; ^2^ Department of Biostatistics The University of Texas MD Anderson Cancer Center Houston Texas USA; ^3^ Department of Nutrition Sciences and Health Behavior, School of Health Professions University of Texas Medical Branch Galveston Texas USA; ^4^ Health Promotion Research Center, College of Medicine The University of Oklahoma Health Sciences Oklahoma City Oklahoma USA

**Keywords:** activity monitors, osteosarcoma, physical activity, step counts

## Abstract

**Purpose:**

Osteosarcoma is the most common primary bone tumor in childhood and adolescence. Many patients face long‐term impairments in their mobility and function after treatment, leading to a decrease in their quality of life. Exercise has been shown to improve functional recovery and improve quality of life in patients with cancer, though data specific to children with osteosarcoma are sparse. Exercise has also been shown to be feasible in patients undergoing chemotherapy, with numerous potential benefits to health and quality of life. To design the most effective exercise interventions for children and adolescents with osteosarcoma, we must first understand the activity patterns in this population throughout the treatment and survivorship continuum.

**Methods:**

In this study, we provided wearable activity trackers to osteosarcoma patients to evaluate physical activity patterns. Inclusion criteria allowed for any age, gender, or stage of treatment (including after treatment completion).

**Results:**

Twenty‐six patients had valid data defined as 3 or more days with more than 10 h of continuous heart rate data. The average steps per day across all treatment stages including post treatment was 3184 ± SD 2552.74, range 0–27,828 steps on treatment days and 4884 ± 2447.30, range 0–22,500 steps on off treatment days. Values for specific treatment periods (neoadjuvant, adjuvant, relapse, off therapy) are presented. Though activity patterns varied widely between patients, all patients except one were below recommended values for daily step counts until after therapy was complete.

**Conclusion:**

Our results suggest that tailoring an exercise program to encourage activity on days when patients receive antineoplastic therapy, and to have the more intensive exercise days well after antineoplastic therapy, may be a good approach. Further research is needed to define interventions to improve physical activity in this population.

**Abbreviations**
AYA: adolescent/young adult
MVPA: moderate—vigorous physical activity
SD: standard deviation

## 1 Introduction

Osteosarcoma is the most common primary bone tumor in childhood and adolescence [1, 2]. The standard of treatment for osteosarcoma is a combination of chemotherapy and surgery, typically a limb‐salvage procedure [3]. However, these interventions result in significantly decreased strength, mobility, and physical function [4, 5]. Physical and occupational therapy help to mitigate these negative effects by improving range of motion, strength, endurance, balance, coordination, and gait [6]. Most patients receive physical and occupational therapy after surgery, with the predominantly functional goals unrelated to increased physical activity or exercise. There is room for improvement in support for increased physical activity in pediatric, adolescent, and young adult (AYA) patients undergoing treatment for osteosarcoma.

There is growing evidence that exercise before surgery, termed “prehabilitation” or “prehab,” can aid the functional recovery after orthopedic surgery [7]. Exercise improves physical function, strength, and fatigue in adult cancer survivors, and is recommended during and after cancer treatment for adults [8, 9, 10, 11]. Similar positive effects of exercise are demonstrated for pediatric and AYA cancer patients [12, 13, 14] and there is a growing interest in implementation of pediatric/AYA‐focused exercise programs in pediatric oncology settings [13]. To optimize the development of exercise interventions or healthy lifestyle programs for pediatric/AYA patients with osteosarcoma, it is important to understand the activity patterns specific to this group throughout the treatment continuum. Self‐reported activity questionnaires have gathered some critical information about behavior patterns in pediatric/AYA patients with cancer [12, 15], and a recent study reported accelerometry‐based 7‐day activity patterns in adolescent cancer survivors with mixed diagnoses [16]. However, there remains a paucity of data measuring the physical activity levels of pediatric/AYA patients with osteosarcoma across neoadjuvant, adjuvant, relapse, and post‐treatment. Patients with osteosarcoma may be unique among pediatric/AYA patients with cancer in terms of their physical activity patterns because they are often restricted to nonweight bearing exercises for the tumor bearing limb during the neoadjuvant treatment phase.

The objective of this study was to determine the level of physical activity of children and AYAs with osteosarcoma in various phases of treatment. The working hypothesis was that patients with osteosarcoma are less mobile than healthy children throughout treatment.

## 2 Methods

### 2.1 Participants

Patients were recruited as part of a large, prospective cohort study that collects physical activity, physical function, nutrition, and quality of life data for a pediatric oncology energy balance data repository [17]. The Institutional Review Board at MD Anderson Cancer Center approved this study (Protocol # 2021–0661). Patients were eligible to enroll if they were 0 or more years of age, had wrists large enough to wear the activity tracking device, were diagnosed with cancer, and were treated at the MD Anderson Child and Adolescent Center or the inpatient Pediatric unit.

### 2.2 Design

Patients with osteosarcoma were recruited, consented, and enrolled on this study from January 2018–July 2024. The study included an “opt in” consent form that allowed patients to select to participate in any or all of the following in a prospective, observational manner: physical activity tracking, food intake reporting, physical function testing, blood vitamin level measurement, or Patient Reported Outcome questionnaires. Demographic and clinical data were collected including patient age, gender, and race. Patients were either newly diagnosed, relapsed, or in remission. Treatment variables including phase of therapy, date of diagnosis, date of surgery, date of relapse, and date of completion of therapy were collected. In this study, “neoadjuvant” included all data collected between diagnosis and local control, “adjuvant” included all data collected after local control while patients were still undergoing treatment for the primary tumor, “relapse” included data collected after a patient had been diagnosed with disease relapse, and “post‐therapy” included data collected after completion of antineoplastic treatment. Inpatient days referred to days in which the patient received a chemotherapy therapy infusion and/or was admitted to the hospital.

For the purpose of the study described in this report, we selected only patients from the broader cohort who had a diagnosis of osteosarcoma and who had consented to physical activity data collection using Fitbit wrist worn activity monitors. The patients received a wrist worn activity monitor (i.e., Fitbit Alta HR or Inspire HR; the models were changed as Fitbit released new models over the 6‐year period in which patients were recruited) at the beginning of the study and were asked to wear the Fitbit for up to 2 years, periodically syncing data to the Fitabase data storage tool. During the first 6 weeks, study team members tracked patient data syncing to the Fitabase platform and provided reminder calls/text when weeks passed without data sync. Based on the recommendations for using Fitbit Data provided by the NIH All of Us Research Program [18], we defined valid wear data as days with 10 or more hours of heart rate data; 3 or more sequential valid wear data days were required for inclusion in the analyses [19]. The study protocol was intentionally written to allow collection of physical activity data for varying time periods between patients. The maximum amount of time for which physical activity data could be collected was 2 years, though many patients opted to wear the physical activity monitor (Fitbit) for less than 2 years.

### 2.3 Outcomes

Here, we present the physical activity patterns, indicated by step count collected using wrist worn activity monitors for extended periods of time (up to 449 days) in a low contact prospective observational design. We report individual data from 26 patients at different stages of neoadjuvant, adjuvant, treatment for relapse, or after completion of treatment.

### 2.4 Statistical Analyses

Patient characteristics were summarized by count and percentage or mean and range. Step counts were summarized by mean, range, standard deviation, and count of days observed.

Physical activity, as steps‐per‐day, was modeled separately for each patient in relation to time by a generalized additive model with penalized spline over time. The model‐predicted trend over time was overlaid on the scatterplot of daily measurements. Vertical lines were added to indicate dates of surgery or relapse/progression, and colored indicator points were added to indicate days where major events occurred, including adjuvant, neoadjuvant, post therapy, inpatient, primary therapy, and relapse regimen. The collected data varied substantially between patients in the timing relative to the treatment phase and the duration of data collection. For this reason, data was not aggregated for group‐wise statistical comparisons but alternatively presented as individual patient data. The intent was not to provide inferential statistics, but rather to provide a summary of the data for each patient. Modeling was performed using R statistical software version 4.4.1. Steps were presented as means with standard deviation (SD) with a range of steps per day.

## 3 Results

A total of 26 patients out of the 30 osteosarcoma patients enrolled on the study had more than 3 valid wear days. They ranged in age from 8 to 27, with the majority being Caucasian (54%) and male (54%). The most common tumor location was the femur (46%) (Table 1). Activity monitor wear duration and data syncing frequency varied. There was a wide range in the number of valid Fitbit wear days between patients, from 8 to 449 days (Table S1).

**TABLE 1:** Patient characteristics (*N* = 26).

Demographics		% of total
Variable	*N*	
Mean Age (16.5)	26	
Age range (8–27)	26	
Race		
Asian	14	7.7%
Caucasian	4	53.8%
African American	3	15.4%
Hispanic	2	11.5%
Middle Eastern	2	7.7%
Sex		
Male	14	53.8%
Female	12	46.2%
Location of tumor		
Femur	12	46.2%
Humerus	4	15.4%
Tibia	4	15.4%
Rib	2	7.7%
Spine	3	11.5%
Mandible	1	3.8%
Patients with Steps during MAP therapy	15	57.7%
Patients with Steps during non‐MAP therapy	15	57.7%
Patients with Steps post therapy	5	19.2%

**FIGURE 1:**
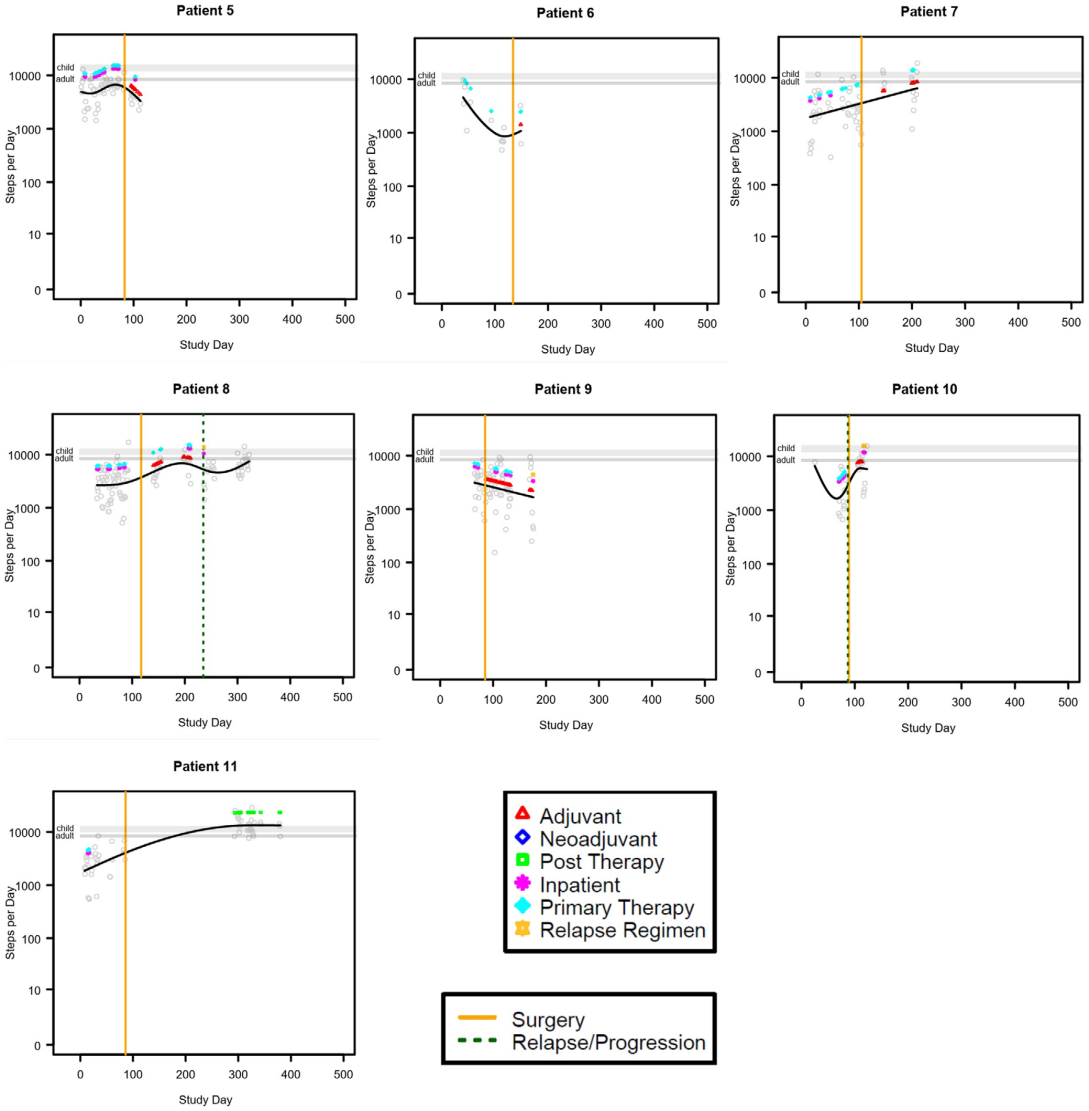
Graphical representation of step patterns for individual patients with data over the span of > 100 days beginning in the neoadjuvant phase. The black solid line represents the penalized spline curve fit to the scatterplot. The gray horizontal lines represent the range of recommended steps per day for children and adults.

**FIGURE 2:**
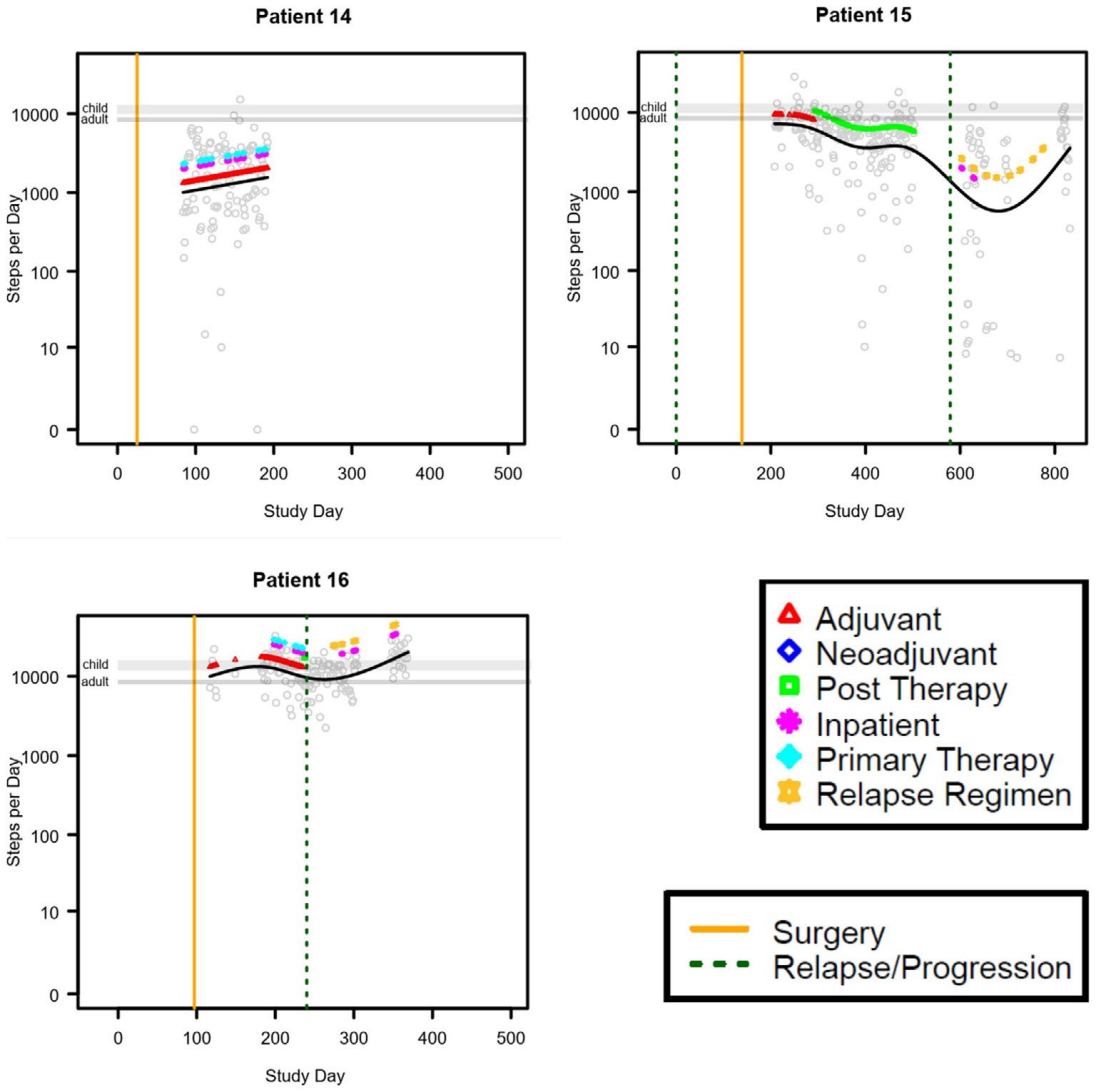
Graphical representation of step patterns for individual patients with data over the span of > 100 days beginning in the adjuvant phase. The black solid line represents the penalized spline curve fit to the scatterplot. The gray horizontal lines represent the range of recommended steps per day for children and adults.

**FIGURE 3:**
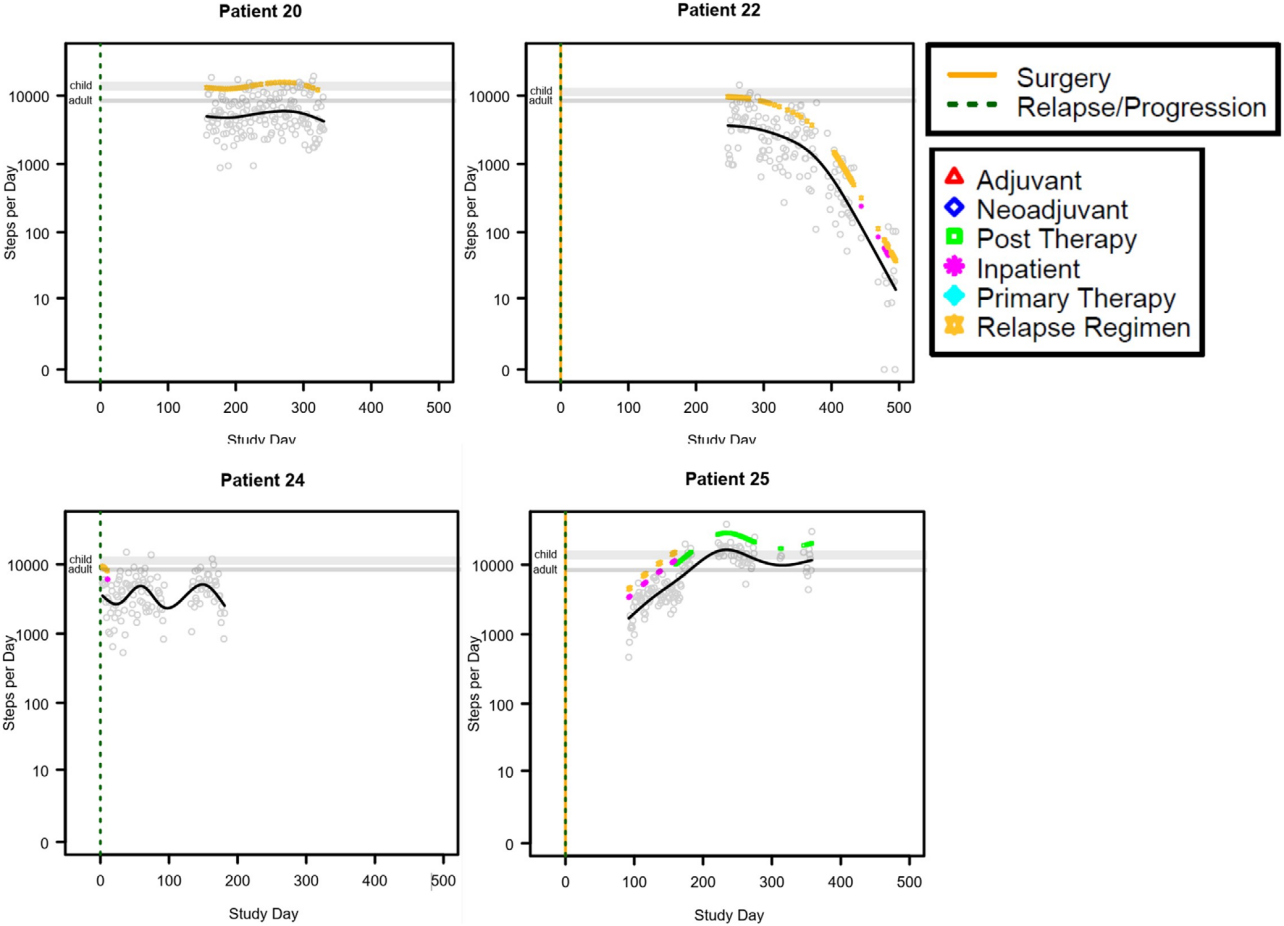
Graphical representation of step patterns for individual patients with data over the span of > 100 days beginning during treatment for relapse or while off therapy. The black solid line represents the penalized spline curve fit to the scatterplot. The gray horizontal lines represent the range of recommended steps per day for children and adults.

We evaluated step counts on valid wear days during neoadjuvant, adjuvant, relapse, and post therapy. There were 11 patients with valid wear days during neoadjuvant treatment, 12 patients with valid wear days during primary adjuvant treatment, 14 during relapse, and 5 during post therapy. Some patients had step counts measured across multiple time periods (Table 2). In general, patients had the least steps during neoadjuvant treatment days. However, given the variability between patients both in timing of data collection relative to antineoplastic treatment plan and in the average number of steps per day, data from each patient is presented individually (Figures 1, 2, 3, Table 2, Figure S1). The average number of steps per day during neoadjuvant therapy was lower on days when patients received antineoplastic therapy (1936 ± 872.4; range 0–6024) than on days when they did not receive therapy (2699 ± 697.50; range 153–10,036. *n* = 11; Figure 1, Table 2, Figure S1). A similar difference was observed during adjuvant therapy and treatment relapse. During adjuvant therapy, the average steps on antineoplastic treatment days was 4320 ± 3812.5 (range 15–19,550, *n* = 9) and 5160 ± 3028.95 (range 0–17,772, *n* = 11, Figure 2, Table 1, Figure S1) during days not on antineoplastic treatment. During treatment for relapse, the average number of steps per day when receiving antineoplastic therapy was 3295 ± 2973.3 (range 0–27,828, *n* = 13) and on days not receiving antineoplastic therapy it was 4214 ± 2467 (range 0–22,150, *n* = 13; Table 1, Figure S1). The highest average number of steps per day occurred during post‐therapy, after all therapy was completed, though the variability remained large between patients (7461 ± 3598.5, range 0–22,500, *n* = 5; Figure 3, Figure S1, Table S2).

**TABLE 2:** Average steps with standard deviations while on antineoplastic therapy and off antineoplastic therapy divided by treatment phase (neoadjuvant, adjuvant, relapse). Post‐therapy patients were not receiving antineoplastic therapy. *N* indicates the number of days with documented steps per patient.

Neoadjuvant						
Patient	Steps on antineoplastic treatment days (mean [range])	SD	*n* (days)	Steps off antineoplastic treatment days (mean [range])	SD	*n* (days)
1	1619 (901–2943)	778.71	7	2299		1
2	1941 (0–6024)	2291.89	6	2239 (153–4325)	2950.04	2
3	1986 (749–3787)	883.88	9	2635 (1016–4022)	1032.5	7
4	1106 (177–3177)	1287.26	7	2356 (282–4592)	1233.83	19
5	2330 (620–4451)	1091.95	19	3344 (1147–6270)	1133.83	33
6	1867 (568–3342)	1427.69	4	1371 (415–4090)	1322.59	8
7	2284 (193–5803)	1603.59	16	3125 (395–10,036)	2431.25	27
8	1868 (628–4595)	1202.07	15	3321 (362–7422)	1745.55	43
9	4169 (2898–5689)	1151.26	4	2584 (605–5147)	1528.26	13
10	1191 (507–2326)	572.47	8	3948 (3183–5164)	732.72	5
11	940 (353–2320)	924.61	4	2474 (266–6860)	1409.51	25

Adjuvant						
Patients	Steps on antineoplastic treatment days (mean [range])	SD	*n* (days)	Steps off antineoplastic treatment days (mean [range])	SD	*n* (days)
4				3483 (3320–3646)	230.51	2
5	3951		1	1725 (951–2579)	477.05	10
6	911 (285–1537)	885.29	2			
7	4336 (1094–8739)	3394	4	9792 (3523–17,722)	4629.79	9
8	7185 (2953–14,423)	4179.22	8	5844 (1662–12,253)	2779.63	15
9	1672 (412–3189)	1026.48	9	3538 (148–9319)	2231.31	37
10				7075 (3915–10,118)	1751.35	10
12	6157 (4762–7672)	1458.7	3	4608 (1135–9752)	2902.62	8
13	956 (271–2081)	667.26	7	994 (85–6716)	1312.79	23
14	1298 (15–5176)	1264.2	34	2746 (0–15,108)	2326.97	75
15				7164 (924–16,321)	3184.17	43
16	12,412 (6638–19,550)	3324.66	15	9787 (1596–16,523)	4320.74	43

Relapse						
Patient	Steps on antineoplastic treatment days (mean [range])	SD	*n* (days)	Steps off antineoplastic treatment day (mean [range])	SD	*n* (days)
8	2345		1	5715 (1409–9555)	1949.36	33
9	423 (384–462)	55.15	2			
10	2381 (1834–3116)	543.58	4	7095 (6661–7528)	613.06	2
15	327 (0–4314)	895.96	39	988 (0–11,797)	2281.11	191
16	11,594 (3385–27,828)	6637.04	14	9786 (1390–22,150)	4731.55	66
17	4265 (1063–6591)	1586.97	11	4663 (646–7534)	1725.85	18
18	4087		1	3182 (939–6113)	1183.66	78
19	2333 (2085–2580)	350.01	2	1443 (0–6649)	1419.11	44
20	5026 (1190–18,317)	3446.4	33	6071 (877–17,147)	3674.48	136
21	520 (158–935)	391.2	3	1991 (280–5172)	1829.8	13
22	1887 (0–8840)	2220.92	85	2579 (109–10,302)	2087.32	72
23				4187 (941–7893)	1448.85	65
24	4982 (3264–5932)	900.04	6	3304 (501–8919)	1835.47	120
25	2662 (468–4654)	1171.38	17	3776 (991–7239)	1482.08	47

Post‐ therapy			
Patient	Steps per day (mean [range])	SD	*n* (days)
11	9759 (5456–22,500)	3800.82	27
15	4771 (0–13,018)	3030.28	176
16	6895 (4121–8272)	1879.84	4
25	12,328 (3068–20,936)	4523.12	80
26	3553 (1371–4923)	1022.9	20

## 4 Discussion

Here, we provide daily step counts for 26 pediatric/AYA patients with osteosarcoma across various phases of treatment and survivorship. Since the timing of data collection relative to the treatment phase and the duration of data collection varied considerably across patients, data for each patient were shown individually. Given the paucity of data on the physical activity patterns of pediatric/AYA patients with osteosarcoma, these data may be useful when defining physical activity recommendations, designing exercise interventions, or generating hypotheses involving physical activity for this population.

The American Academy of Pediatrics recommends 60 min of physical activity for school age children daily, which equates to 13,000 to 15,000 steps/day in boys and 11,000 to 12,000 steps/day in girls. For adolescents regardless of sex, 60 min of moderate to vigorous physical activity (MVPA) is 10,000 to 11,700 steps [20]. Normative average steps per day are similar to recommendations; normative values for boys is 12,000 to 16,000 steps/day, for girls is 10,000 to 13,000 steps/day, and for adolescents, steps/day steadily decrease until approximately 8000–9000 steps/day are observed in 18‐year‐olds [20]. The recommended activity level for adults is 150 min of MVPA per week (~8000–11,000 steps/day), though normative steps/day for healthy adults are 7000–13,000 [21].

Our results support our hypothesis that osteosarcoma patients undergoing treatment are less active than healthy children and AYAs. Of the 26 patients included in this study, only 4 had average step counts within the recommended ranges at any point of the observed period, though several more had some days within the recommended range. Most of the patients had step counts below the recommended values while receiving treatment but increased the number of steps as they progressed beyond neoadjuvant therapy. This may be due to reduced cancer burden and comorbid symptoms such as pain, fractures, or nonweight bearing restrictions as patients progressed through treatment. Most patients also had a decrease in their steps postsurgery, as they likely experienced some immobility postoperatively related to the surgical procedure, time needed to heal postsurgery, and adjusting to crutches in the nonweight bearing time period. Patients in all phases of therapy appeared to have more steps on days when they did not receive antineoplastic treatment as compared to days that they did receive antineoplastic treatment. This may be because treatment‐related side effects hinder physical activity, or because of the lack of opportunity to be physically active while hospitalized. Our results suggest that interventions are needed to increase physical activity in patients with osteosarcoma during treatment.

Exercise interventions for pediatric patients with osteosarcoma are safe and feasible [22], and have been shown to partially mitigate the significant physical, psychosocial, and socioeconomic impairments that can occur due to decreased mobility and function [5, 6]. In children with various cancers, exercise has been associated with an increase in function as well as an increase in quality of life [23]. In adults with cancer, exercise has been shown to improve quality of life and physical function, and recently, to increase survival for some cancer types [24, 25, 26, 27, 28, 29, 30, 31]. In adult patients with cancer, lower numbers of steps per day are associated with risk of severe surgical complications [32], and increased steps per day are associated with increased survival in advanced cancer patients with multiple cancer etiologies [33].

Our results suggest that there is wide variability in the activity levels of pediatric and AYA patients with osteosarcoma, and many of the benefits of exercise may be missed by patients in this population unless intentional effort is made to support more physical activity and exercise. Most patients in this cohort had much lower physical activity on the day(s) of chemotherapy infusion, with increased activity in the time between infusions. Since patients receive chemotherapy in clinical settings, one could leverage the patients' attendance on site to provide access to supervised physical activity (e.g., treadmill, walking, or stationary bicycle while wearing chemotherapy backpacks, etc.). We also hypothesize that chemotherapy‐related side effects may be driving the sedentary behavior on these inactive days. This also presents an opportunity for improvement (e.g., adequate chemotherapy induced nausea/vomiting management with prioritization for nonsedating medications, as aligned with Children's Oncology Group recommendations). Providers should encourage patients to remain active during and after therapy with guidance on safe forms of exercise and referral to Physical and Occupational Therapy as needed. A limitation of this study is that the act of wearing an activity tracker could have positively influenced the activity level of the patients leading to a biased result. Major limitations of this study include the small sample size, the lack of a local ‘healthy’ reference population instead of national standards, and the limited number of days that accelerometry data was acquired for each patient over the study period. Due to the small sample size, we were not able to compare patient groups based on phase of therapy or type of therapy or draw firm conclusions regarding predictors of step counts. Furthermore, we were unable to correlate step count with clinical outcomes. Future research should evaluate physical activity in a larger sample of osteosarcoma patients to assess predictors of activity and correlations between physical activity and clinical outcomes.

## Author Contributions

Elysia R. Cohen, Karen Moody, Maria C. Swartz, and Keri Schadler: conceptualization. Clark Andersen, Michael C. Robertson, and Alakh Rajan: methodology. Clark Andersen and Michael C. Robertson: software. Elysia R. Cohen, Theresa Honey, Sandra Lugo, Grace Waterman, and Keri Schadler: investigation. Clark Andersen: formal analysis. Karen Moody, Maria C. Swartz, and Keri Schadler: supervision. Elysia R. Cohen: visualization. Karen Moody, Maria C. Swartz, and Keri Schadler: project administration. Elysia R. Cohen: writing – original draft. Clark Andersen, Karen Moody, Maria C. Swartz, Alakh Rajan, Theresa Honey, Sandra Lugo, Grace Waterman, Keri Schadler, and Michael C. Robertson: writing – review and editing.

## Funding

This work was supported by the University of Texas MD Anderson Cancer Center.

## Conflicts of Interest

The authors declare no conflicts of interest.

## Supporting information


**Figure S1:** Graphical representation of step patterns for individual patients with data over the span of < 100 days. The black solid line represents the penalized spline curve fit to the scatterplot. The gray horizontal lines represent the range of recommended steps per day for children and adults.

**Table S1:** Total days of fitbit wear per patient arranged in ascending order by patient ID (left columns) or number of steps from least to greatest (right columns).
**Table S2:** The average steps per day and average number of days of fitbit wear per treatment phase (neoadjuvant, adjuvant, relapse, and post therapy) while on and off antineoplastic therapy.

## Data Availability

The data that support the findings of this study are available on request from the corresponding author. The data are not publicly available due to privacy or ethical restrictions.
