# Supplementary figures and images for: Physical Activity in Osteosarcoma Patients During and Post Therapy: A Single Site Prospective Observational Study
Source: Cancer Med. 2026 Feb 26;15(3):e71674. doi: 10.1002/cam4.71674 (PMC12945699; doi:10.1002/cam4.71674)

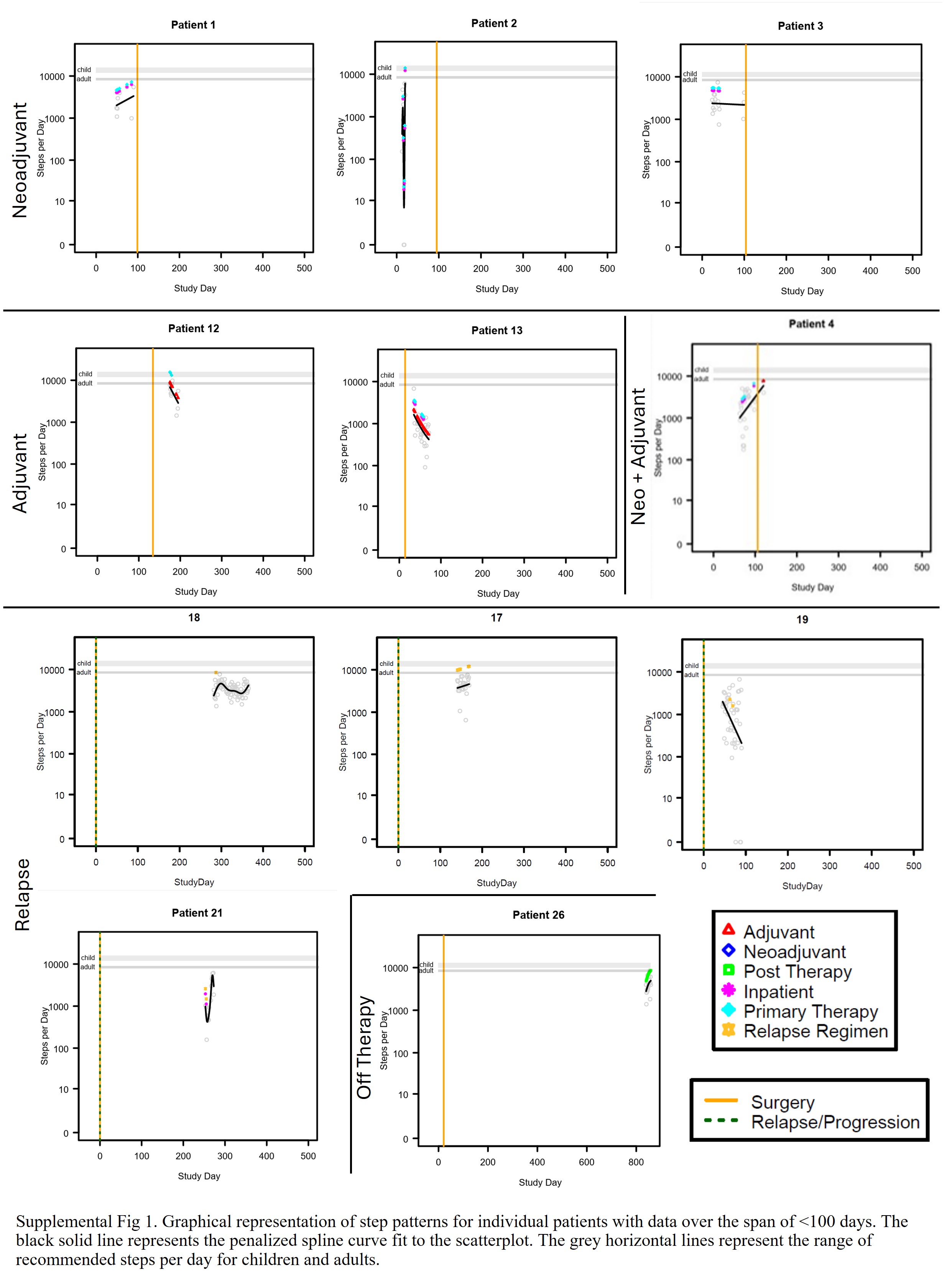

Supplement: Supplementary file 1 — Figure S1: Graphical representation of step patterns for individual patients with data over the span of < 100 days. The black solid line represents the penalized spline curve fit to the scatterplot. The gray horizontal lines represent the range of recommended steps per day for children and adults. [file CAM4-15-e71674-s002.jpg]
