# Supplementary material for: Physical Activity in Osteosarcoma Patients During and Post Therapy: A Single Site Prospective Observational Study
Source: Cancer Med. 2026 Feb 26;15(3):e71674. doi: 10.1002/cam4.71674 (PMC12945699; doi:10.1002/cam4.71674)
Supplement: Supplementary file 2 — Table S1: Total days of fitbit wear per patient arranged in ascending order by patient ID (left columns) or number of steps from least to greatest (right columns). Table S2: The average steps per day and average number of days of fitbit wear per treatment phase (neoadjuvant, adjuvant, relapse, and post therapy) while on and off antineoplastic therapy. [file CAM4-15-e71674-s001.docx]

Supplemental Table 1

| **Total Days of Fitbit Wear** | | | | |
| --- | --- | --- | --- | --- |
| Patient | Days |  | Days | Patient |
| 1 | 8 |  | 8 | 1 |
| 2 | 8 |  | 8 | 2 |
| 3 | 16 |  | 11 | 12 |
| 4 | 28 |  | 14 | 6 |
| 5 | 63 |  | 16 | 3 |
| 6 | 14 |  | 16 | 21 |
| 7 | 56 |  | 20 | 26 |
| 8 | 115 |  | 28 | 4 |
| 9 | 65 |  | 29 | 17 |
| 10 | 29 |  | 29 | 10 |
| 11 | 56 |  | 30 | 13 |
| 12 | 11 |  | 46 | 19 |
| 13 | 30 |  | 56 | 11 |
| 14 | 109 |  | 56 | 7 |
| 15 | 449 |  | 63 | 5 |
| 16 | 142 |  | 65 | 9 |
| 17 | 29 |  | 65 | 23 |
| 18 | 79 |  | 79 | 18 |
| 19 | 46 |  | 109 | 14 |
| 20 | 169 |  | 115 | 8 |
| 21 | 16 |  | 126 | 24 |
| 22 | 157 |  | 142 | 16 |
| 23 | 65 |  | 144 | 25 |
| 24 | 126 |  | 157 | 22 |
| 25 | 144 |  | 169 | 20 |
| 26 | 20 |  | 449 | 15 |

Supplemental Table 2

|  | **Steps on antineoplastic treatment days (mean (range))** | **SD** | **Average days (range)** | **Steps off antineoplastic treatment days (mean (range))** | **SD** | **Average days (range)** |
| --- | --- | --- | --- | --- | --- | --- |
| Neoadjuvant | 1936 (0-6,024) | 872.40 | 9 (4-19) | 2,699 (153- 10,036) | 697.50 | 17 (1-43) |
| Adjuvant | 4,320 (15-19,550) | 3812.50 | 9 (1-34) | 5,160 (0- 17,722) | 3028.95 | 25 (2-75) |
| Relapse | 3,295 (0-27,828) | 2973.33 | 17 (1-85) | 4,214 (0- 22,150) | 2467.05 | 68 (2-191) |
| Post therapy |  |  |  | 7,461 (0- 22,500) | 3598.52 | 61 (4-176) |
